# Innovations on Dementia Advocacy among Students of a Medical University in Taiwan

**DOI:** 10.1093/geroni/igab046.2962

**Published:** 2021-12-17

**Authors:** Tsuann Kuo

**Affiliations:** Chung Shan Medical University, Chung Shan Medical University / Taichung City, Taichung, Taiwan (Republic of China)

## Abstract

In 2017, Taiwan established Dementia Action Plans 2.0 to respond to the World Health Organization's call to increase dementia awareness and support for dementia carers. However, efforts have not yet been made to educate and increase dementia literacy on the younger generations. This paper addressed the outcomes to increase knowledge and information about dementia and caregiver resources to university students. 93 students participated in a two-day workshop on dementia literacy, followed by two months of advocacy in Taichung City, Taiwan. Students were divided into 14 advocacy groups and the outcome reports were categorized qualitatively using content analysis. The results showed that students were from nine departments and over one-quarter having a dementia loved one in the family. Four innovative categories were developed, including (1) dementia literacy for students and the public; (2) dementia friendly action plans; (3) dementia caregiver’s advocacy and (4) reducing dementia stereotypes. Highlights included students using social media to promote dementia literacy, face-to-face experiences to inform public education, dementia education on early onset dementia and using diverse bio-psycho-social angels to evaluate dementia. Students expanded advocacy to many social media, innovations and target areas, including Facebook, Instagram, Google spreadsheet, stickers, postcards, illustration of children’s books and public announcement. This paper revealed that younger generations used many advocacy methods that were thinking outside of the box. In conclusion, dementia is no longer an elder’s business but young adults can bring technological, inter-generational and cultural innovations into fulfilling the goals of Dementia Global Action Plans.

